# Mechanism of Smilax china L. in the treatment of intrauterine adhesions based on network pharmacology, molecular docking and experimental validation

**DOI:** 10.1186/s12906-024-04414-4

**Published:** 2024-04-05

**Authors:** Tingting Shi, Chuqi Hou, Yongzhen Duan, Yuliang Li, Wenqin Liu, Peixian Huang, Yuhua Zhou, Shanshan Yu, Luyao Song

**Affiliations:** 1 Department of Pharmacy, Zhujiang Hospital, Southern Medical University, #253 Industrial Avenue Zhong, Guangzhou, 510280 Guangdong China; 2 Clinical Pharmacy Center, Nanfang Hospital, Southern Medical University, Guangzhou, 510515 China

**Keywords:** Intrauterine adhesion, Smilax china L., Astilbin, Molecular docking, Network pharmacology, PI3K/AKT

## Abstract

**Background:**

Smilax china L. (SCL) is a traditional herbal medicine for the potential treatment of intrauterine adhesion (IUA). However, the mechanisms of action have not yet been determined. In this study, we explored the effects and mechanisms of SCL in IUA by network pharmacology, molecular docking and molecular biology experiments.

**Methods:**

Active ingredients and targets of SCL were acquired from TCMSP and SwissTargetPrediction. IUA-related targets were collected from the GeneCards, DisGeNET, OMIM and TTD databases. A protein‒protein interaction (PPI) network was constructed by Cytoscape 3.9.1 and analysed with CytoHubba and CytoNCA to identify the core targets. The DAVID tool was used for GO and KEGG enrichment analyses. Furthermore, molecular docking was employed to assess the interaction between the compounds and key targets. Finally, the mechanisms and targets of SCL in IUA were verified by cellular experiments and western blot.

**Results:**

A total of 196 targets of SCL were identified, among which 93 were related to IUA. Topological and KEGG analyses results identified 15 core targets that were involved in multiple pathways, such as inflammation, apoptosis, and PI3K/AKT signalling pathways. Molecular docking results showed that the active compounds had good binding to the core targets. In vitro experiments showed that astilbin (AST), a major component of SCL, significantly reduced TGF-β-induced overexpression of fibronectin (FN), activation of the PI3K/AKT signalling pathway and the expression of downstream factors (NF-κB and BCL2) in human endometrial stromal cells, suggesting that AST ameliorates IUA by mediating the PI3K/AKT/NF-κB and BCL2 proteins.

**Conclusions:**

AST, a major component of SCL, may be a potential therapeutic agent for IUA. Moreover, its mechanism is strongly associated with regulation of the PI3K/AKT signalling pathway and the downstream NF-κB and BCL2 proteins. This study will provide new strategies that utilize AST for the treatment of IUA.

**Supplementary Information:**

The online version contains supplementary material available at 10.1186/s12906-024-04414-4.

## Introduction

Intrauterine adhesion (IUA), also known as Asherman syndrome, is a uterine disorder characterized by pelvic or intrauterine scar formation and adhesions. IUA can result in irregular menstruation, habitual miscarriage, and even infertility [1]. Approximately 66–77.5% of patients with severe IUA develop infertility [2]. IUA is sometimes induced by inflammation and infection but is more often elicited by mechanical injuries resulting in fibrosis, such as hysteroscopy and dilation and curettage (D&C), which has been reported to be the primary cause of IUA [3–7]. The incidence of IUA is about 30% following intrauterine instrumentation, and this rate is increasing with the popularization of hysteroscopy and the increase in abortions [8]. However, there are few effective therapies for IUA. Even for the preferred treatment, transcervical resection of adhesion (TACR), the recurrence rate after surgery is as high as 62.5% for severe IUA [9, 10]. Therefore, the search for an effective drug to prevent or treat IUA is an essential task.

Smilax china L. (SCL), also known as “Baqia” or “Jingangteng” in China, is a Chinese herbal medicine with anti-inflammatory, anticancer, and antioxidant properties that promotes blood circulation and removes blood stasis [11–15]. SCL has shown significant effects in gynaecological diseases [16–18]. It has been suggested that SCL may inhibit inflammatory factors and tissue fibrosis [17]. Consistent with our previous research, studies have shown that ingredients of SCL can alleviate uterine inflammation and fibrosis, which implies that SCL may be effective in alleviating IUA [16]. However, how these compounds interact with IUA disease targets to disrupt the related signalling pathways remains unclear.

Network pharmacology is a multidisciplinary discipline based on bioinformatics and systems biology. It is based on the systematic mining of data and analysis by software to predict the relationship between drug targets and diseases. It is now extensively available for exploring the therapeutic targets and mechanisms of herbal components in various diseases.

**Fig. 1:**
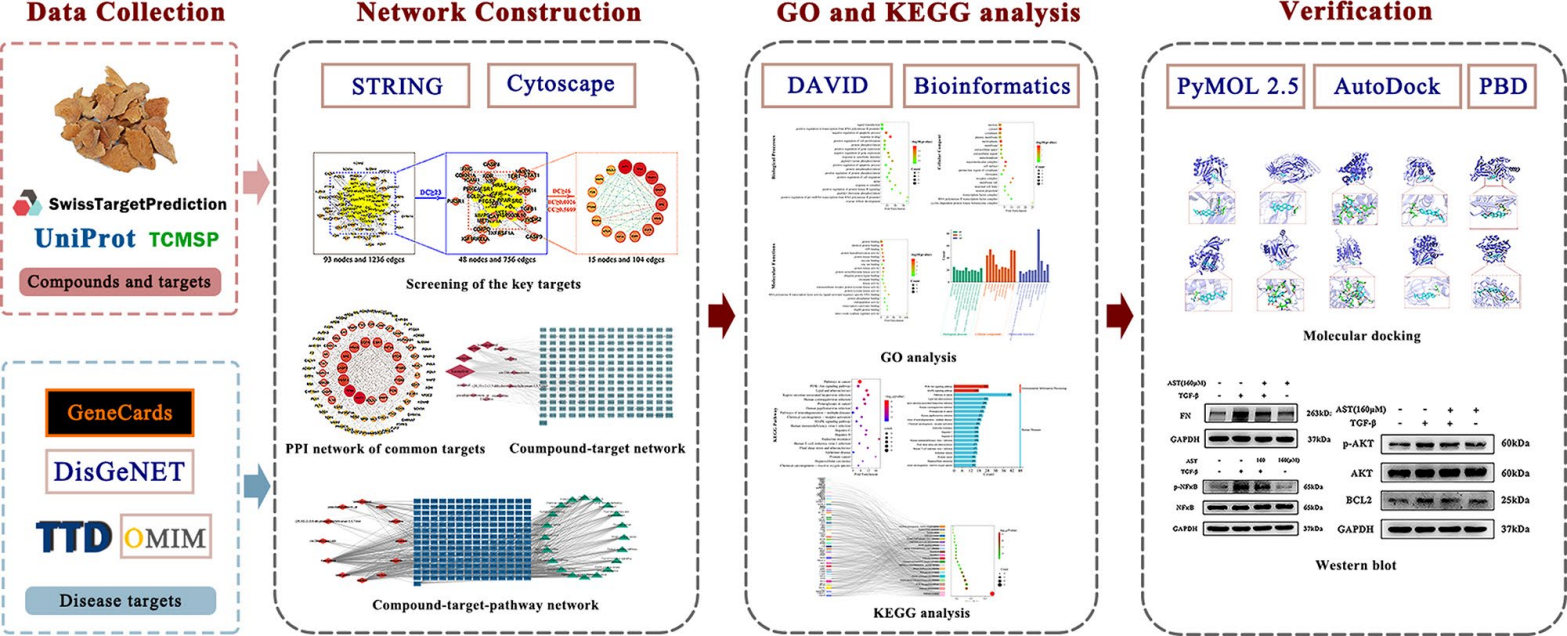
Flowchart of the network pharmacology study of SCL for the treatment of IUA

Therefore, in this work, network pharmacology and molecular docking were used to systematically investigated the potential targets, mechanisms and bioactive ingredients of SCL against IUA. Next, experimental validation was utilized to determine the mechanism of SCL for the treatment of IUA. The research process of this work is shown in Fig. 1. We sought to explore the potential molecular connection between SCL and IUA and the possible therapeutic mechanism of SCL for the treatment of IUA.

## Materials and methods

### Material collection

#### Collecting active ingredients and targets of SCL

Chemical ingredients and targets of SCL were screened for in the Traditional Chinese Medicine Systems Pharmacology Database (TCMSP, https://old.tcmsp-e.com/tcmsp.php) and the SwissTargetPrediction database (http://swisstargetprediction.ch/). These herbal pharmacology databases provide access to the active compounds, targets and pharmacokinetics of herbal medicines, including drug-likeness (DL) and oral bioavailability (OB). First, the active ingredients and targets of SLC were initially evaluated. The threshold for screening according to TCMSP database were OB ≥ 30 and DL ≥ 0.18. Second, the 2D structures were downloaded from the PubChem database (https://pubchem.ncbi.nlm.nih.gov/) based on the component names, and the predicted targets with probability*>0 were selected after importing their molecular structures using the SwissTargetPrediction database (http://swisstargetprediction.ch/). To standardize the target information, the screened proteins were standardized in the UniProt database (https://www.uniprot.org) to normalize the names. Finally, the potential targets of SCL were merged, and duplicate values were removed.

#### Screening of targets for IUA

IUA-related targets were obtained by using “intrauterine adhesion” and “intrauterine adhesions” as keywords and searching four databases, including the GeneCards database (https://www.genecards.org/), OMIM (https://www.omim.org/), DisGeNET (https://www.disgenet.org/), and TTD (https://db.idrblab.net/ttd/). First, the IUA target data in GeneCards were collected, and the median of the relevance score was calculated. Then, the targets were filtered base on the condition of being greater than the median (median = 1.409075). Furthermore, the targets from the 4 databases were combined by Excel to remove duplicates. Finally, the list of targets of IUA was established.

### Network construction

#### Active ingredient-target network construction for SCL

Cytoscape is an analysis software for the visualization of molecular interaction networks, allowing for the analysis of active ingredient and drug target interactions. The drug components and targets collected above were visualized and managed using Cytoscape 3.9.1, and an interaction network was constructed. In this network, each component or target represents a node, and interconnections are represented by lines.

#### Constructing a network of potential targets

To confirm the intersecting targets of SCL and IUA, we used the bioinformatics visualization cloud platform (http://www.bioinformatics.com.cn/static/others/jvenn/example.html) to obtain intersection targets for SCL and IUA and generate a Venn diagram. The above targets were imported into the String database (https://string-db.org) using the selection “Homo Sapiens” with a default confidence level of greater than or equal to 0.4; the results were saved in TSV format. Finally, we constructed protein‒protein interaction (PPI) networks to visualize the targets with Cytoscape 3.9.1, with larger degree values represented by larger nodes and darker colours representing larger target influence.

The core targets were filtered using the plugin CytoNCA in Cytoscape 3.9.1. The plugin provides three centralities to assess, which include degree centrality (DC), closeness centrality (CC), and betweenness centrality (BC) [19, 20]. We chose DC/BC/CC greater than the median as the screening criteria for visual analysis. Briefly, the steps were performed as follows: First, targets with DC values greater than 2 times the median were selected. Finally, targets with BC and CC greater than the median were selected as key targets for further molecular docking. Cytohubba in Cytoscape is a plugin used to visualize key targets and subnetworks in PPI networks by selecting the top 10 as key targets. The MCODE function of Cytoscape 3.9.1 was used to perform a modular analysis of the PPI network, setting the cut-off point as follows: K-core > 2, max depth > 100, and node score > 0.2 [21].

#### Construction of component-target-pathway networks

For visualization and elucidation of the intricate connections between compounds, targets, and pathways, we constructed and analysed compound-target-pathway in Cytoscape 3.9.1. The method is the same as 2.2.2.

### GO and KEGG enrichment analyses

The common targets were entered into the DAVID database (https://david.ncifcrf.gov/summary.jsp), the species selected was Homo sapiens, and the screening cut-offs were *P* < 0.05 and FDR < 0.05. The analysis results of Gene Ontology (GO) and Kyoto Encyclopedia of Genes and Genomes (KEGG) signalling pathway enrichment were obtained. The top 10 targets were selected for visual analysis using the bioinformatics website (https://www.bioinformatics.com.cn/).

### Molecular docking

The top six components were identified from the compounds and docked to the ten key targets. In brief, the docking process was performed as follows. First, the files of the molecular structure (in mol2 format) were downloaded from the TCMSP, and then AutoDockTools 1.5.7 was used to perform hydrogenation of the structures. Second, the target structures were obtained from the RCSB Protein Data Bank Protein (https://www.rcsb.org/), imported into PyMOL 2.5.5 (https://pymol.org/2/), dehydrated, hydrogenated and separation of ligand. Docking grid boxes were constructed at the active site of each protein using AutoDockTools 1.5.7. Next, the target and active molecules were molecularly docked and assessed free binding energies with the number of genetic algorithm runs for 50 times. The docking parameters were selected as default. Other parameters were number of generations for picking worst individual = 10, docking nice level = 20. We selected the binding pattern with the lowest binding energy. Finally, PyMOL 2.5.5 was used for the visualization and analysis of interactions. The final access date is May 19th, 2023.

### Cell culture

Immortalized human endometrial stromal cells (T-HESCs) were purchased from the American Type Culture Collection (CRL-4003; ATCC). T-HESCs were maintained in growth media (DMEM/F12 without phenol red + 10% FBS + 1% insulin, transferrin, selenium (ITS) + 100 U/mL penicillin and 100 mg/mL streptomycin) at 37 °C in a suitable atmosphere of 5% CO_2_. Astibin (AST) was purchased from Shanghai Tauto Biotech Co., Ltd. (Shanghai, China). AST was dissolved in DMSO at a concentration of 160 mM. To verify the effect of AST on endometrial fibrosis, AST (concentration of 160 µM) was added 1 h prior to 48 h of TGF-β1 induction.

### Cell counting Kit-8 assay

T-HESCs were inoculated in 96-well plates at a density of 8 × 10^3^, with five replicate wells in each group. After AST intervention for 24 h/48 h, 10 µl of CCK8 (Glpbio) solution was added to each well and the samples were incubated for 3–4 h at 37 °C. OD values were measured at 450 nm using an enzyme marker, and survival rates were calculated.

### Western blot analysis

Proteins were extracted from T-HESCs using RIPA lysis buffer, and the protein concentration was quantified by BCA. After separation by SDS-PAGE gel, the proteins were transferred to PVDF membranes. After 2 h or 15 min of blocking with 5% skim milk or fast blocking solution, the membranes were incubated with antibodies against FN (sc-8842; Santa Cruz), p-AKT (4060; Cell Signaling Technology), AKT (4691; Cell Signaling Technology), NF-κB p65 (T55034; Abmart), p-NF-κB p65 (sc-136,548; Santa Cruz), BCL2 (ab182858; Abcam) and GAPDH (60004-1-Ig; Proteintech) at 4 °C overnight. Following three washes with TBST, the membranes were incubated with HRP-conjugated Affinipure Goat Anti-Rabbit IgG (1:10000) for 1 h at room temperature and then visualized by chemiluminescence. The densities of protein were determined by ImageJ.

### Statistical analysis

Data from the experiments were expressed as the mean ± SD. The numerical data satisfied the criteria for parametric tests. Thus, differences between groups were analysed by one-way Analysis of Variance. These data were analysed using SPSS version 26.0 analysis software, and graphs were generated by GraphPad Prism 8. *P* < 0.05 indicated statistical significance.

## Results

### Active compounds and potential targets of SCL against IUA

**Fig. 2:**
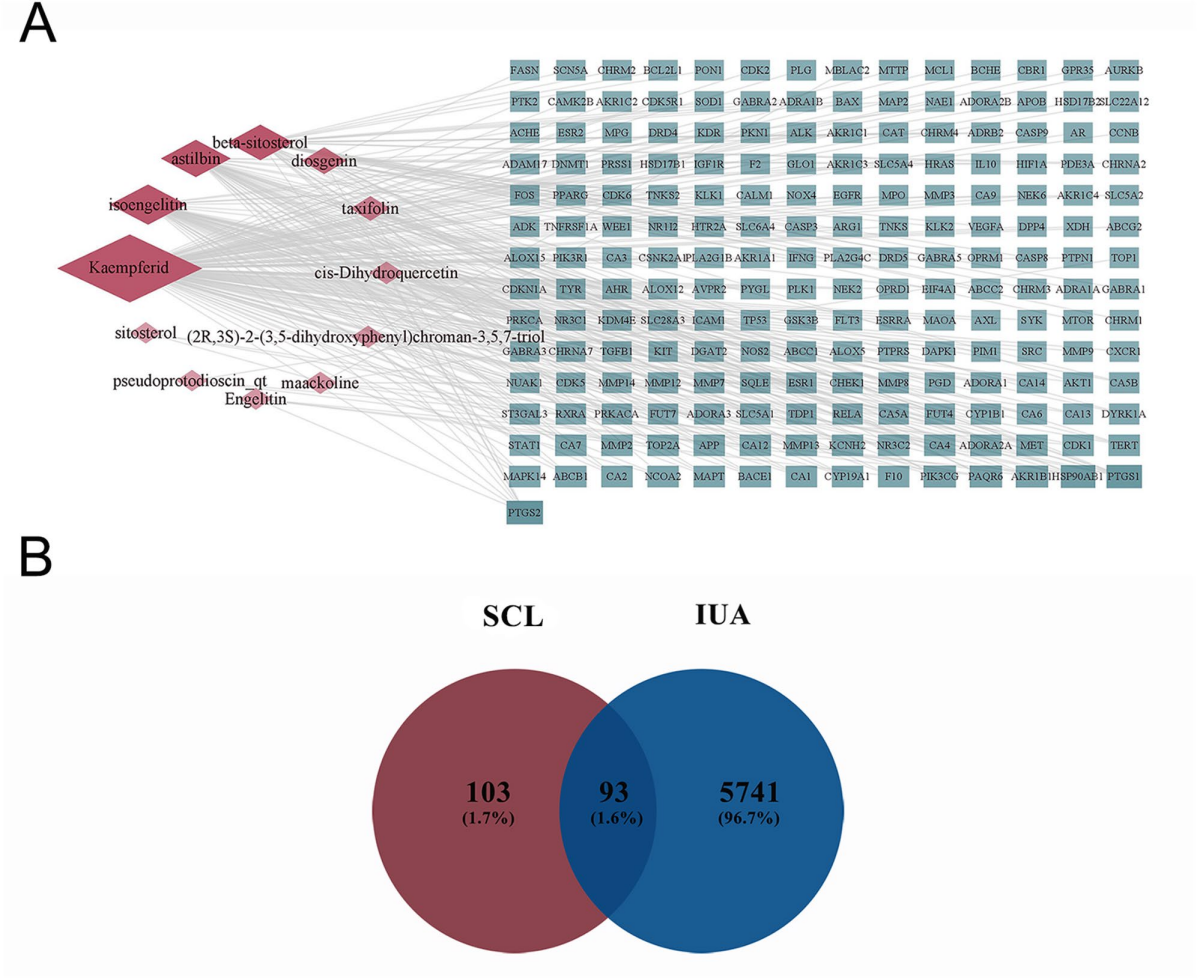
Collecting active compounds and target. (**A**) compound-target network of SCL. Red diamonds represent the active compounds of SCL. Green rectangles are the targets. The colour depth and node size are positively correlated with the degree value. (**B**) Venn diagram of 93 common targets from IUA and SCL

Twelve chemical compounds in SCL were acquired from TCMSP and SwissTargetPrediction (See Supplementary Table 1, Additional File 1). A total of 197 targets of SCL active compounds and their targets information were collected by TCMSP database (See Supplementary Table 2, Additional File 1). By applying Cytoscape 3.9.1, the chemical compounds and 197 targets were created a compound-target network which consisted of 259 nodes and 1273 edges (Fig. 2A). A network analysis showed that kaempferid (degree: 120), isoengelitin (degree: 48), astilbin (degree: 41), beta-sitosterol (degree: 37), diosgenin (degree: 16), and taxifolin (degree: 11) were key nodes.

There were 3772 IUA targets initially obtained from GeneCards, and 1886 targets with a score greater than or equal to the median (≥ 1.41) were selected as potential targets. In addition, we merged disease targets acquired from DisGeNET, OMIM, TTD and GeneCards and deleted duplicates, yielding 5834 IUA targets (See Supplementary Table 3, Additional File 1). To identify potential targets of SCL for the treatment of IUA, the SCL targets and IUA targets were imported into the online bioinformatics website. Common targets are shown in the Venn diagram (Fig. 2B). The intersection of SCL targets with IUA disease targets included 93 (1.6%) key targets of SCL for IUA treatment (See Supplementary Table 4, Additional File 4).

### PPI network of common targets

**Fig. 3:**
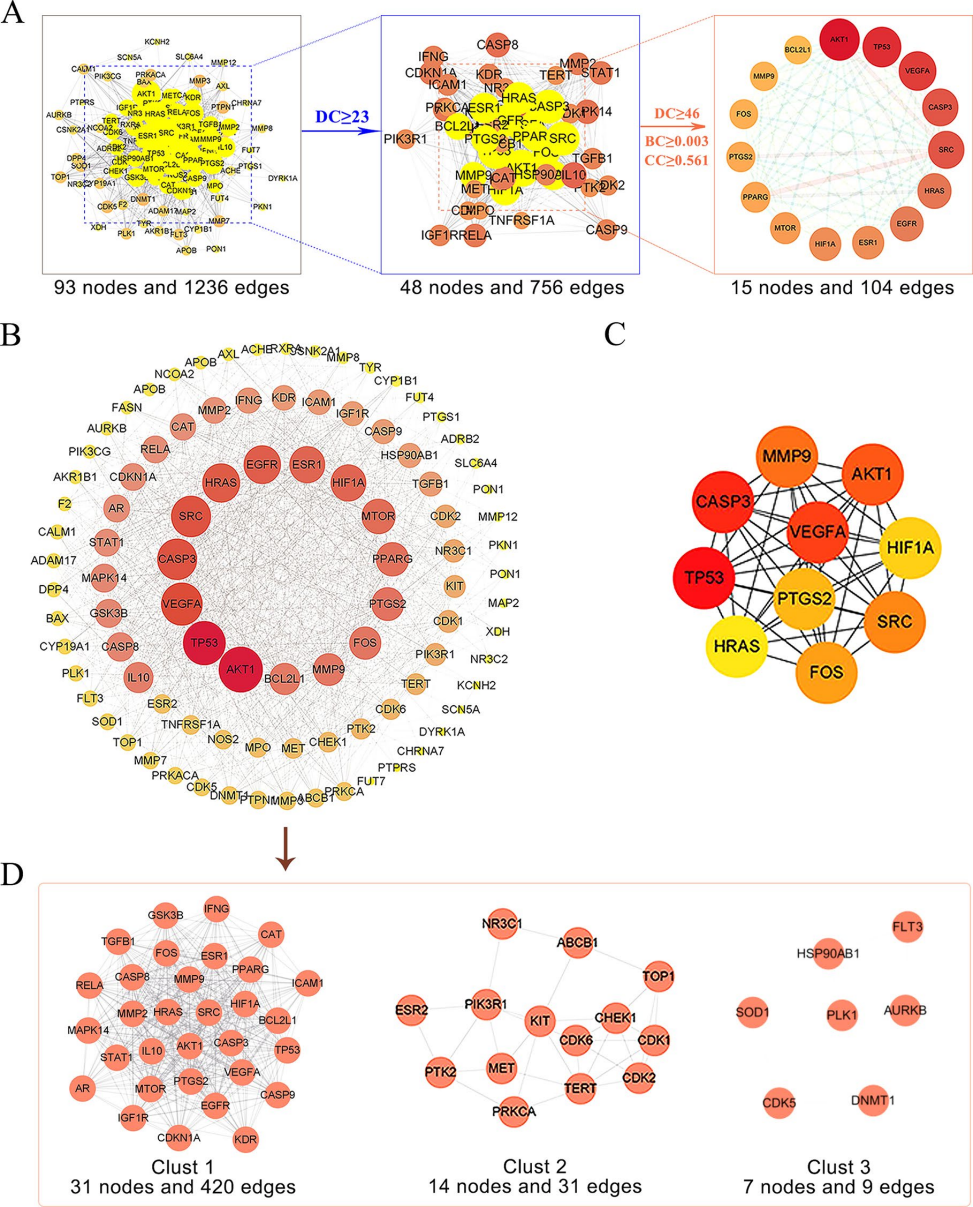
Screening the potential targets by protein-protein interaction (PPI) for IUA. (**A**) Filtering process for topology analysis. The 15 key targets were obtained via DC, BC and CC. (**B**) Established a network of core and non-core targets. The core targets are in the inner circle. (**C**) The 10 hub genes were determined using CytoHubba. The change from yellow to red indicates that the degree value is gradually getting larger. (**D**) Cluster analysis was conducted using MCODE

To identify the potential targets of SCL in IUA, 93 intersecting targets were inputted into the STRING website to establish a PPI network, which was consisted of 93 nodes and 1236 edges. Core targets were identified with a screening threshold of the median CC, BC and 2-fold median DC values, which were 46, 0.561 and 0.003 for DC, CC and BC, respectively. The screening criteria for key targets is greater than the threshold. After the above screening, fifteen core targets in 93 common targets were determined through network analysis (Table 1; Fig. 3A). A network of core and noncore targets was established (Fig. 3B). In this network, the node size and the colour depth were proportional to the degree of targeting. The 10 hub genes were selected from MCC top 10 using the CytoHubba tool in Cytoscapse (Fig. 3C). MCODE was used to perform a clustering analysis to generate a linked subnetwork and assign these targets to the three clusters (Fig. 3D).

**Table 1:** Basic information of 15 core targets

NO.	Uniprot ID	Gene symbol	Protein name		Degree
1	P31749	AKT1	RAC-alpha serine/threonine-protein kinase		72
2	P04637	TP53	Cellular tumor antigen p53		69
3	P15692	VEGFA	Vascular endothelial growth factor A		67
4	P12931	SRC	Proto-oncogene tyrosine-protein kinase Src		64
5	P42574	CASP3	Caspase 3		64
6	P01112	HRAS	HRas Proto-Oncogene, GTPase		60
7	P00533	EGFR	Epidermal growth factor receptor		59
8	P03372	ESR1	Estrogen receptor		56
9	Q16665	HIF1A	Hypoxia-inducible factor 1-alpha		56
10	P42345	MTOR	Serine/threonine-protein kinase mTOR		53
11	P37231	PPARG	Peroxisome proliferator activated receptor gamma		51
12	P35354	PTGS2	Prostaglandin G/H synthase 2		50
13	P01100	FOS	Transcription factor AP-1		49
14	P14780	MMP9	Matrix metalloproteinase-9		48
15	Q07817	BCL2L1	Apoptosis regulator Bcl-2		47

### GO enrichment analysis

**Fig. 4:**
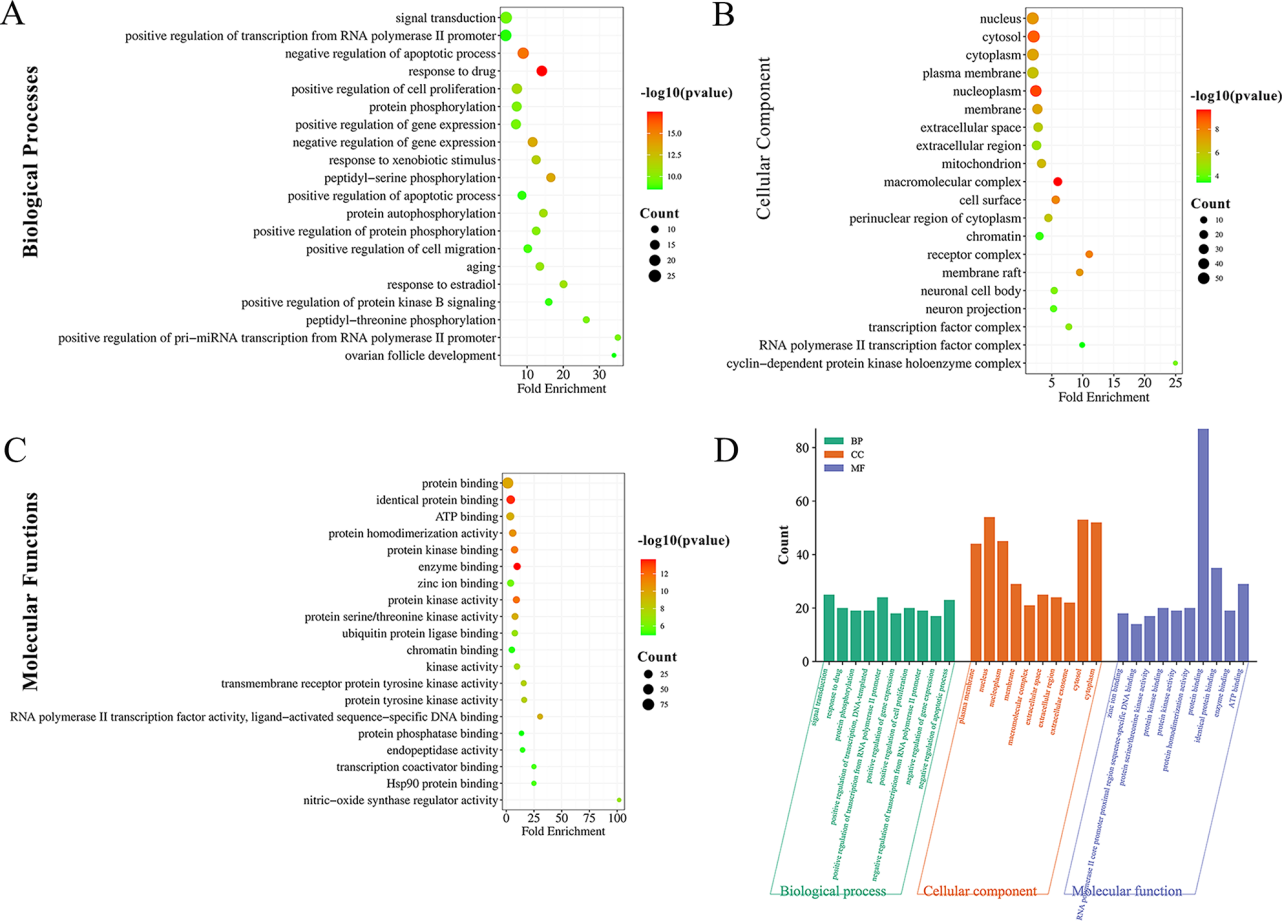
Results of Gene ontology (GO) enrichment analysis. (**A**) Enrichment dot bubble diagrams for the analysis of biological processes for 93 targets. The x- and y-axis represent fold enrichment and GO terms, respectively. The colour and dot sizes are the *P* value and the count. (**B**) Enrichment dot bubble diagrams for the analysis of cellular components for 93 targets. (**C**) Enrichment dot bubble diagrams for the analysis of molecular function. (**D**) Histogram for the top 10 items of BP, CC, MF

For further investigation of the potential mechanism of SCL in the treatment of IUA, 93 intersecting targets were submitted to the DAVID database for GO enrichment analysis. Using *P* ≤ 0.05 as the cut-off condition, a total of 365 items were identified, including 260 biological processes (BP), 34 cellular components (CC) and 71 molecular functions (MF) (See Supplementary Tables 5–7, Additional File 5–7). The top 20 items in each of the three categories were visualized in the bioinformatics online mapping tool (Fig. 4A-C). The top 10 terms with the lowest P values in each category (total 30) are shown in the bar charts (Fig. 4D). The top five BP terms in GO analysis were mainly enriched in signal transduction, positive regulation of transcription from RNA polymerase II promoter, negative regulation of apoptotic process, response to drug, and positive regulation of cell proliferation. Highly enriched CC terms were nucleus, cytosol, cytoplasm, and plasma membrane. In addition, MF terms included protein binding, identical protein binding, ATP binding, protein homodimerization activity, and protein kinase binding.

### KEGG pathway analysis

**Fig. 5:**
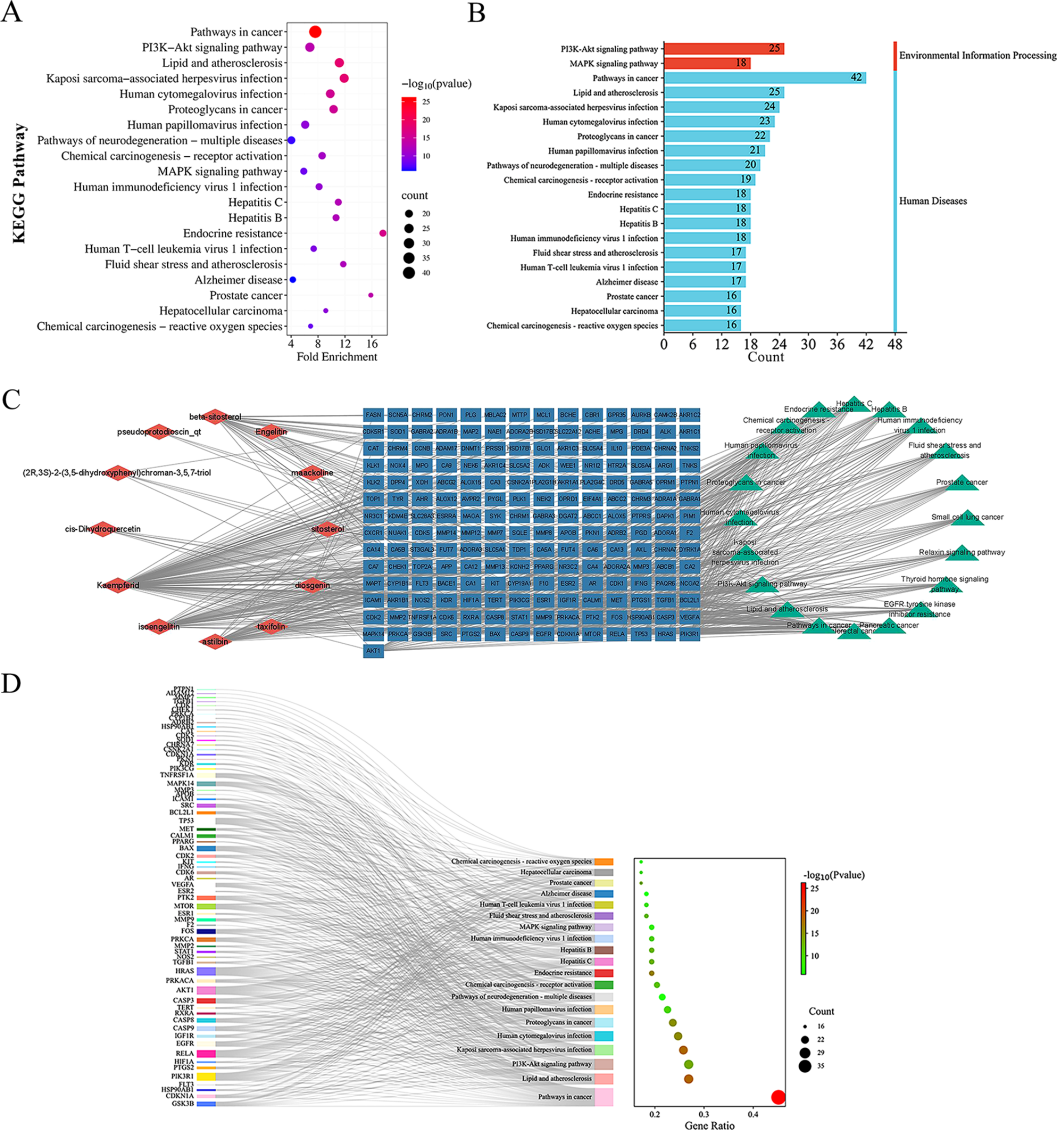
Results of KEGG pathway analysis and compound-target-pathway network. (**A**) The results of the top 20 KEGG pathway are shown in a bubble chart. (**B**) Categorization and summary of the top 20 KEGG pathways. (**C**) Component-target-pathway interaction network construction based on 93 common targets. The network was shown by red diamonds, blue rectangles and green triangles, indicating the components, targets and pathways respectively. (**D**) Sankey chart of SCL potential therapeutic targets and KEGG pathways. The left rectangle nodes represent the potential therapeutic targets and the right rectangles represent the KEGG pathway. The size of the rectangle is directly proportional to the number of lines, indicating the level of connection

**Fig. 6:**
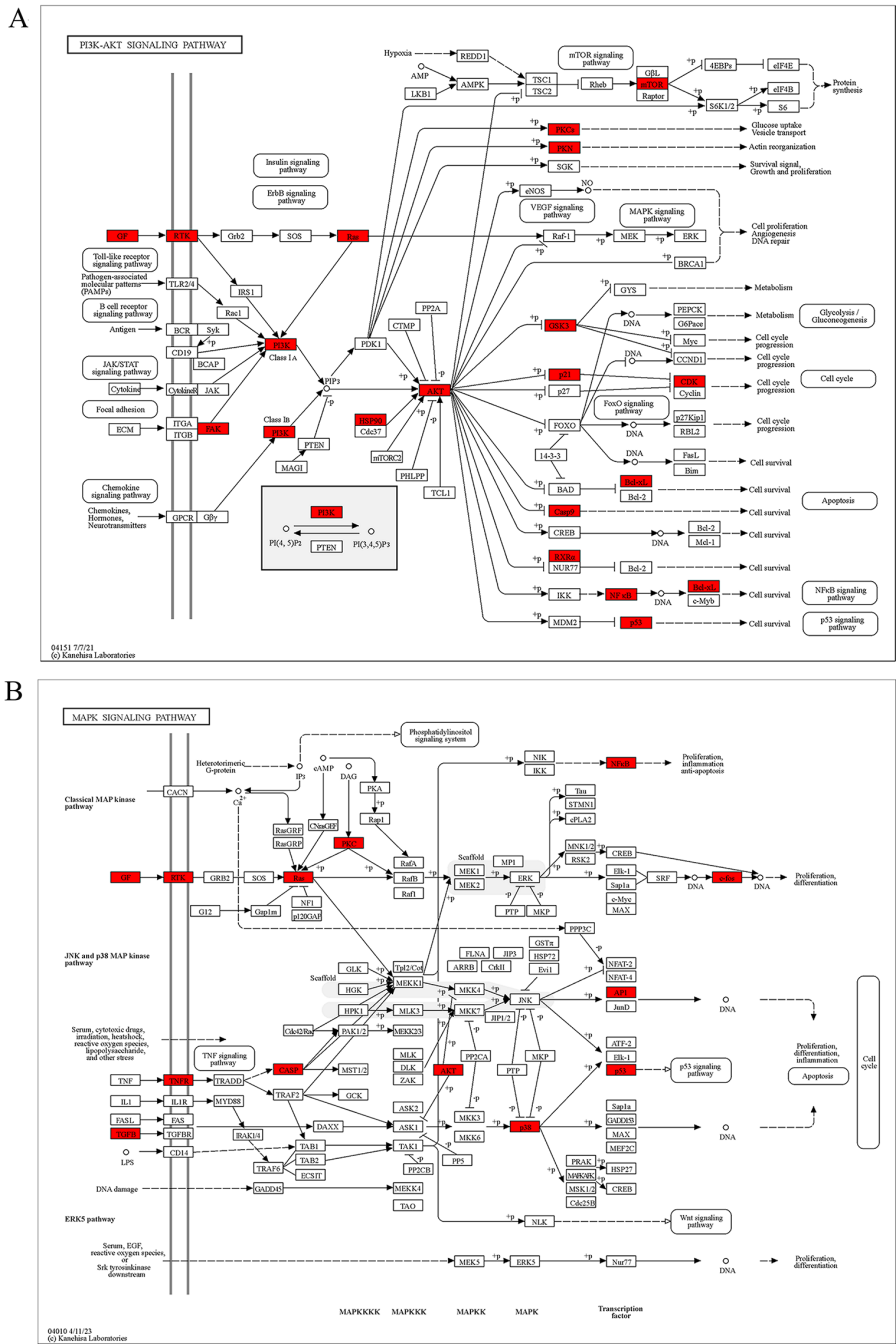
Distribution of common targets in the two most associated pathways. (**A**) Location of common targets in the PI3K-AKT signalling pathway. (**B**) Location of common targets in the MAPK signalling pathway. The rectangle represents the gene name, and the red rectangles are able to reflect the linkage of the common targets of SCL for IUA treatment in the signalling pathway

The 93 common genes were assessed by KEGG analysis (with *P* < 0.05) enriched in 148 pathways (See Supplement Table 8, Additional File 8). The top 20 KEGG items were screened and categorized based on gene count (Table 2; Fig. 5A and B). We constructed a compound-target-pathway network by selecting the top 20 pathways based on the *P* values and gene counts from the KEGG enrichment results, as well as the relevant compounds and targets (Fig. 5C). The network included 262 nodes and 704 edges. The top five targets, AKT, HRAS, RELA, PIK3 and TP53, were related to 17, 16, 14, 17 and 14 pathways, respectively. The linkage between common genes and pathways constitutes a Sankey diagram (Fig. 5D). The high level of enrichment of the PI3K/AKT and MAPK signalling pathways suggests that SCL may treat IUA through both pathways. The KEGG Pathway Database (https://www.genome.jp/kegg/pathway.html) was used to visualize these two pathways (Fig. 6A and B).

**Table 2:** KEGG analysis results of common targets

ID	Term	Count	Fold Enrichment	Gene	P Value
hsa05200	Pathways in cancer	42	7.60	GSK3B, CDKN1A, HSP90AB1, FLT3, PIK3R1, PTGS2, HIF1A, RELA, EGFR, IGF1R, CASP9, CASP8, RXRA, TERT, CASP3, AKT1, PRKACA, HRAS, TGFB1, NOS2, STAT1, MMP2, PRKCA, FOS, F2, MMP9, ESR1, MTOR, PTK2, ESR2, VEGFA, AR, CDK6, IFNG, KIT, CDK2, BAX, PPARG, CALM1, MET, TP53, BCL2L1	5.57E-27
hsa05417	Lipid and atherosclerosis	25	11.17	GSK3B, HSP90AB1, SRC, PIK3R1, RELA, ICAM1, CASP9, CASP8, RXRA, CASP3, AKT1, APOB, HRAS, MMP3, PRKCA, FOS, MAPK14, MMP9, PTK2, TNFRSF1A, BAX, PPARG, CALM1, TP53, BCL2L1	5.19E-19
hsa05167	PI3K-Akt signaling pathway	25	6.78	GSK3B, CDKN1A, HSP90AB1, FLT3, PIK3R1, RELA, EGFR, PIK3CG, IGF1R, CASP9, RXRA, KDR, AKT1, HRAS, PRKCA, MTOR, PTK2, VEGFA, CDK6, KIT, CDK2, PKN1, MET, TP53, BCL2L1	5.37E-14
hsa01522	Kaposi sarcoma-associated herpesvirus infection	24	11.88	GSK3B, CDKN1A, STAT1, SRC, PIK3R1, FOS, PTGS2, MAPK14, HIF1A, MTOR, PIK3CG, RELA, ICAM1, TNFRSF1A, VEGFA, CASP9, CASP8, CDK6, CASP3, BAX, AKT1, CALM1, HRAS, TP53	8.08E-19
hsa05163	Human cytomegalovirus infection	23	9.82	GSK3B, CDKN1A, SRC, PRKCA, PIK3R1, PTGS2, MAPK14, EGFR, MTOR, RELA, PTK2, TNFRSF1A, VEGFA, CASP9, CASP8, CDK6, CASP3, BAX, AKT1, CALM1, PRKACA, HRAS, TP53	3.36E-16
hsa05205	Proteoglycans in cancer	22	10.31	CDKN1A, TGFB1, SRC, MMP2, PRKCA, PIK3R1, MAPK14, HIF1A, ESR1, MMP9, EGFR, MTOR, PTK2, IGF1R, VEGFA, CASP3, KDR, AKT1, PRKACA, HRAS, MET, TP53	6.83E-16
hsa05215	Human papillomavirus infection	21	6.09	GSK3B, CDKN1A, STAT1, PIK3R1, PTGS2, EGFR, MTOR, RELA, PTK2, TNFRSF1A, VEGFA, CASP8, CDK6, TERT, CASP3, CDK2, BAX, AKT1, PRKACA, HRAS, TP53	8.21E-11
hsa01521	Chemical carcinogenesis - receptor activation	19	8.61	HSP90AB1, SRC, CHRNA7, PRKCA, PIK3R1, FOS, ADRB2, ESR1, EGFR, MTOR, RELA, ESR2, VEGFA, AR, RXRA, CYP1B1, AKT1, PRKACA, HRAS	2.94E-12
hsa04151	Endocrine resistance	18	17.64	CDKN1A, SRC, MMP2, PIK3R1, FOS, MAPK14, ESR1, MMP9, EGFR, MTOR, PTK2, ESR2, IGF1R, BAX, AKT1, PRKACA, HRAS, TP53	6.78E-17
hsa05160	Hepatitis C	18	11.01	GSK3B, CDKN1A, STAT1, PIK3R1, EGFR, RELA, TNFRSF1A, CASP9, CASP8, RXRA, CDK6, IFNG, CASP3, CDK2, BAX, AKT1, HRAS, TP53	2.37E-13
hsa05222	Hepatitis B	18	10.67	CDKN1A, TGFB1, STAT1, SRC, PRKCA, PIK3R1, FOS, MAPK14, MMP9, RELA, CASP9, CASP8, CASP3, CDK2, BAX, AKT1, HRAS, TP53	4.00E-13
hsa05161	Human immunodeficiency virus 1 infection	18	8.15	PRKCA, PIK3R1, FOS, MAPK14, MTOR, RELA, PTK2, TNFRSF1A, CASP9, CASP8, CASP3, CHEK1, CDK1, BAX, AKT1, CALM1, HRAS, BCL2L1	3.22E-11
hsa05418	Fluid shear stress and atherosclerosis	17	11.75	HSP90AB1, SRC, MMP2, PIK3R1, FOS, MAPK14, MMP9, RELA, PTK2, ICAM1, TNFRSF1A, VEGFA, IFNG, KDR, AKT1, CALM1, TP53	4.97E-13
hsa05212	Prostate cancer	16	15.84	GSK3B, CDKN1A, HSP90AB1, MMP3, PIK3R1, MMP9, EGFR, MTOR, RELA, IGF1R, CASP9, AR, CDK2, AKT1, HRAS, TP53	3.30E-14
hsa05207	EGFR tyrosine kinase inhibitor resistance	15	18.24	GSK3B, SRC, PRKCA, PIK3R1, EGFR, MTOR, IGF1R, VEGFA, AXL, KDR, BAX, AKT1, HRAS, MET, BCL2L1	3.53E-14
hsa04919	Small cell lung cancer	15	15.66	CDKN1A, NOS2, PIK3R1, PTGS2, RELA, PTK2, CASP9, RXRA, CDK6, CASP3, CDK2, BAX, AKT1, TP53, BCL2L1	3.21E-13
hsa05170	Thyroid hormone signaling pathway	15	11.91	NCOA2, GSK3B, STAT1, SRC, PRKCA, PIK3R1, HIF1A, ESR1, MTOR, CASP9, RXRA, AKT1, PRKACA, HRAS, TP53	1.53E-11
hsa04926	Relaxin signaling pathway	15	11.17	TGFB1, NOS2, SRC, MMP2, PRKCA, PIK3R1, FOS, MAPK14, MMP9, EGFR, RELA, VEGFA, AKT1, PRKACA, HRAS	3.69E-11
hsa05210	Pancreatic cancer	14	17.69	CDKN1A, TGFB1, STAT1, PIK3R1, EGFR, MTOR, RELA, VEGFA, CASP9, CDK6, BAX, AKT1, TP53, BCL2L1	5.03E-13
hsa05165	Colorectal cancer	13	14.52	GSK3B, CDKN1A, TGFB1, PIK3R1, FOS, EGFR, MTOR, CASP9, CASP3, BAX, AKT1, HRAS, TP53	5.19E-11

### Molecular docking

For validation of the network pharmacology findings, we utilized molecular docking to predict the binding of the screened components to targets. Given the level of degree and the PPI network analysis, six effective compounds and ten targets were evaluated by molecular docking. The ten targets and ID are AKT1 (3os5), TP53 (8bc8), VEGFA (4gls), CASP3 (2xyp), RELA (6tan), HIF1 (3hqu), MTOR (3wf7), MMP9 (4wzv), BCL2 (3zln), EGFR (5gnk). Information on the pharmacokinetics for six compounds was collected from ADMETlab (https://admet.scbdd.com/) and SwissAMDE (http://www.swissadme.ch/index.php). (See Supplementary Table 9, Additional File 9).

**Fig. 7:**
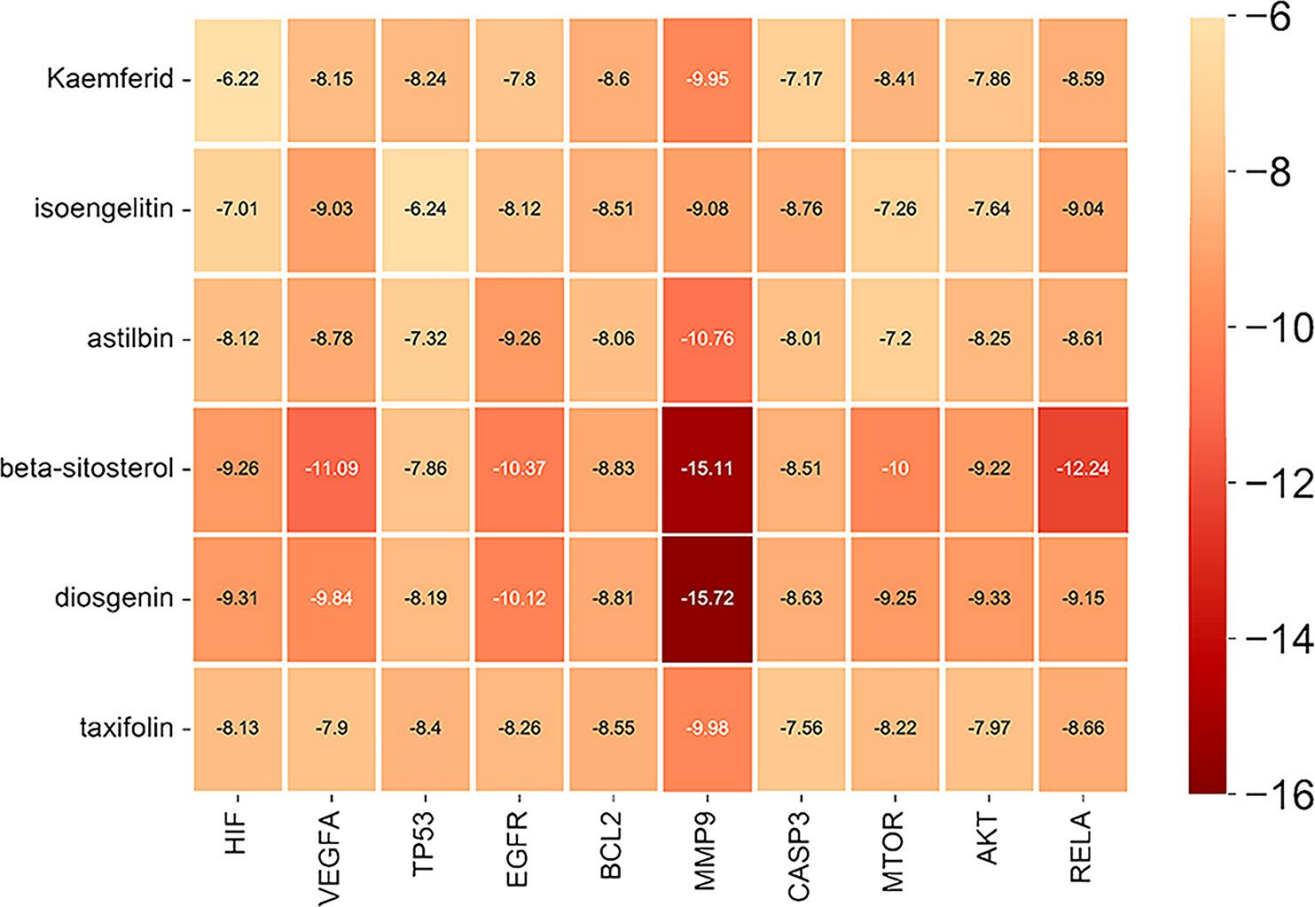
Heat map of the binding energies for molecular docking. The redder the colour, the lower the binding energies, which indicates a more stable binding of the target to the component

**Fig. 8:**
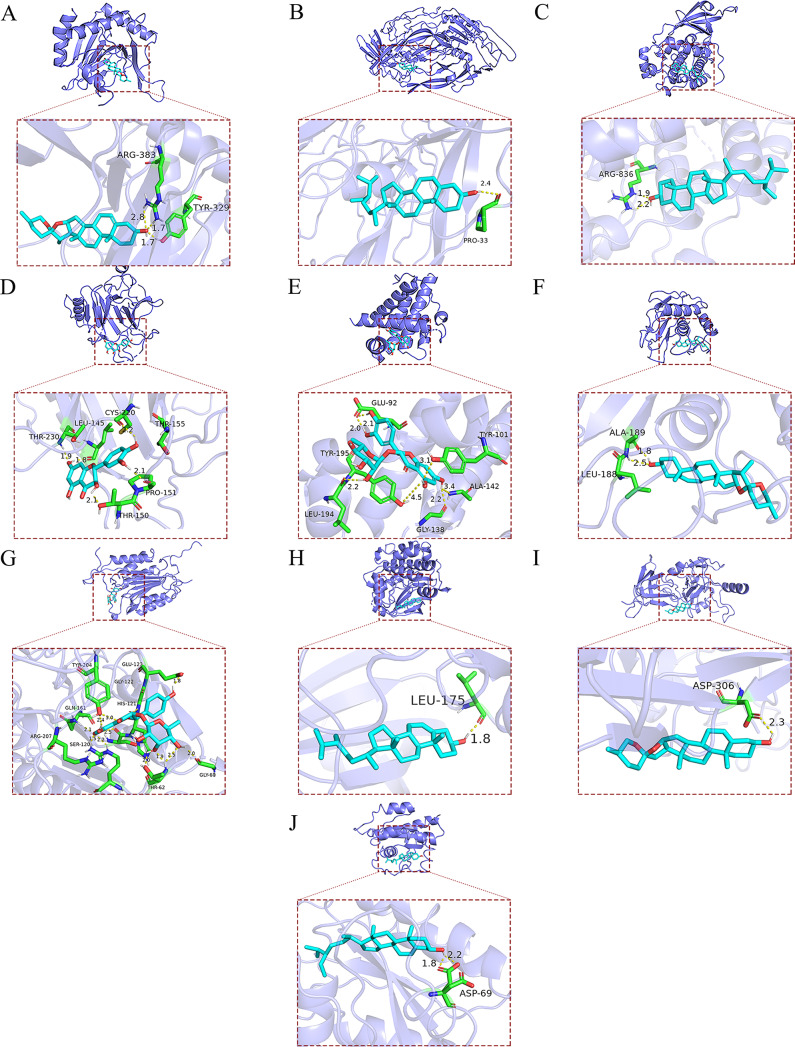
Visualization of target and component molecular docking results. (**A**) HIF-kaemferid (**B**) VEGFA-beta-sitosterol (**C**) TP53-taxifolin (**D**) EGFR-beta-sitosterol (**E**) BCL2-AST (**F**) MMP9-diosgenin (**G**) CASP3-isogenlitin (**H**) MTOR-beta-sitosterol (**I**) AKT-diosgenin (**J**) RELA-beta-sitosterol

The results of the binding energy were illustrated in Fig. 7. The free binding energies from − 6.22 to 15.72 kcal/mol, implying fine and stable binding. Molecular docking of the lowest binding energies of identified targets and components was visualized in PyMOL 2.5.5 (Fig. 8).

### AST alleviates TGF-β-induced fibrosis in T-HESCs by decreasing PI3K/AKT/NF-κB

**Fig. 9:**
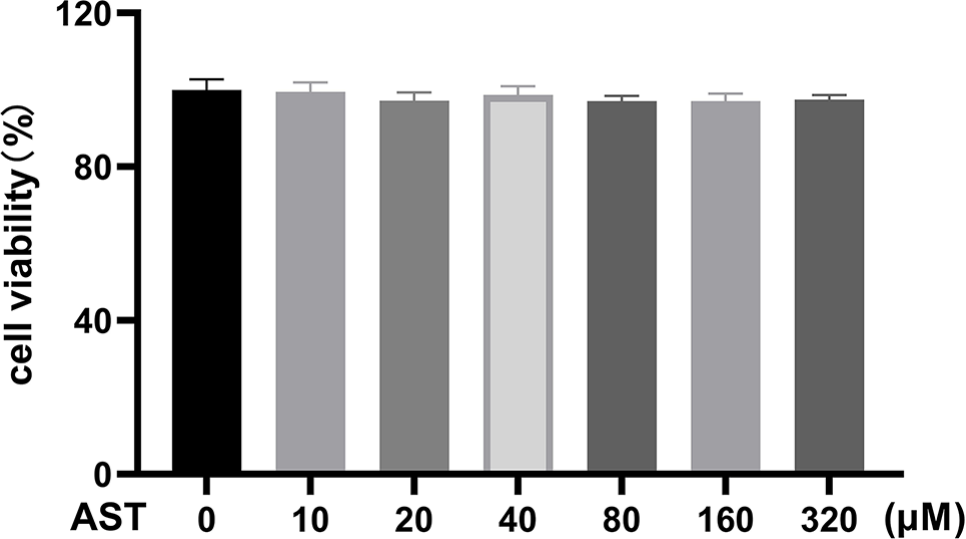
The cell viability by CCK8 assay. T-HESCs were incubated with different concentrations of AST (10, 20, 40, 80, 160, 320 µM) for 48 h. AST indicates astilbin

**Fig. 10:**
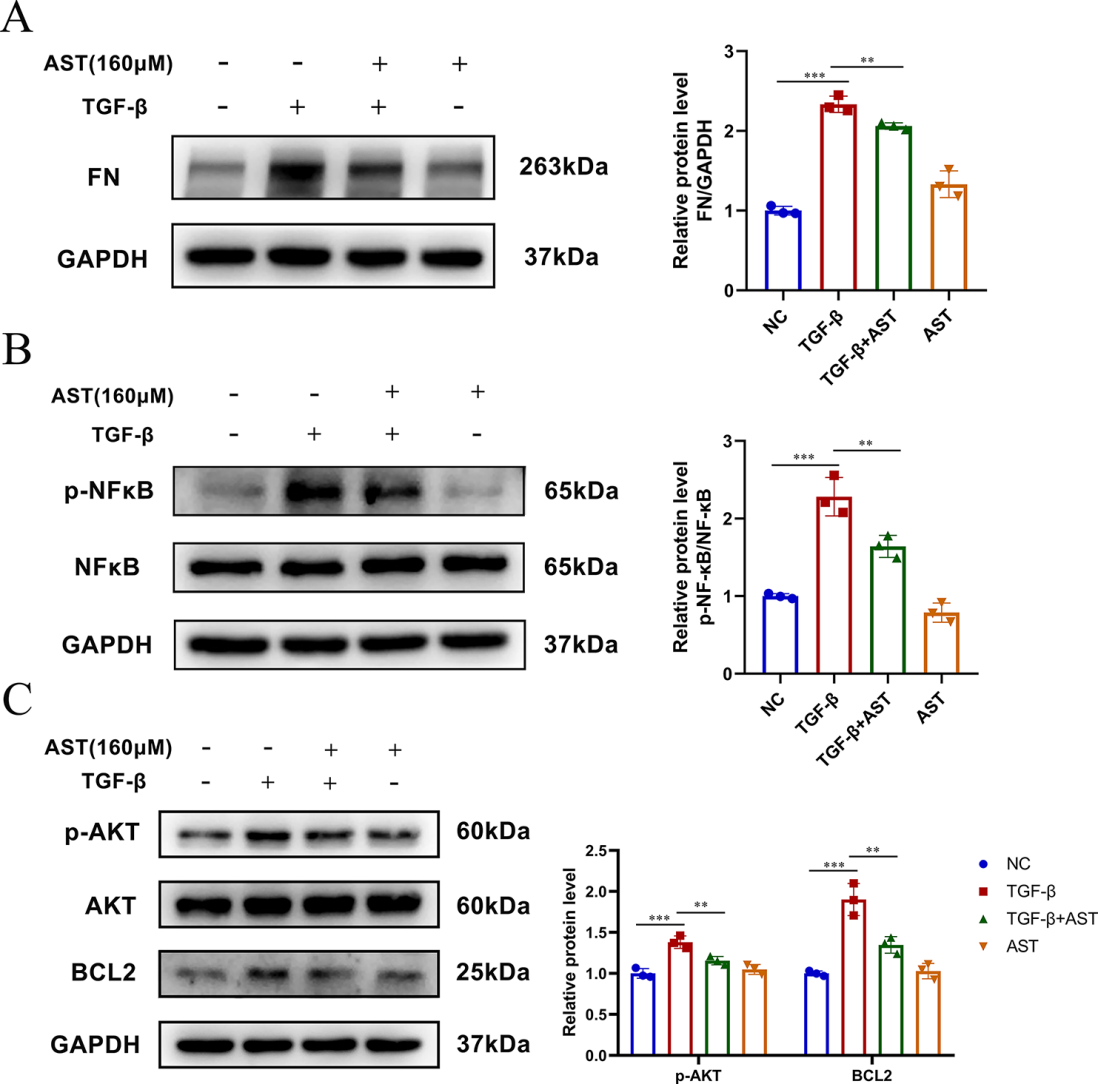
AST affects TGF-β-induced fibrosis in T-HECSs through the PI3K/AKT/NF-κB pathway. T-HESCs were incubated with TGF-β (10ng/ml) for 48 h prior to action with AST for 1 h (*n* = 3). (**A**) The expression and quantification of FN by western blot analysis. (**B**) The expression and quantification of p-NF-κB/NF-κB by western blot analysis. (**C**) The expression and quantification of p-AKT/AKT and BCL2 by western blot analysis. Analysis of data was done using One-way ANOVA or Dennett’s T3. AST indicates astilbin. *P < 0.05, **P < 0.01, ***P < 0.001. The original blots are presented in Additional File 10 Figure S1A-C

Next, we investigated the molecular mechanisms by which AST regulates IUA. AST is a major active component of SCL. Furthermore, the above results suggest that SCL may alleviate IUA by altering the PI3K/AKT signalling pathway. NF-κB (RELA) and PI3K/AKT are involved in the pathogenesis of IUA. BCL2 is downstream of NF-κB. Therefore, we investigated whether AST has an effect on AKT and NF-κB activation in T-HESCs. First, a CCK8 viability assay showed that AST had no effect on cell viability under a 320 μm concentration (Fig. 9). Thus, 160 µM was determined as the treatment concentration for AST. Western blot analysis showed that the levels of FN expression in T-HESCs were significantly decreased by AST, compared with model group (TGF-β). This result suggested that AST alleviates endometrial fibrosis. Moreover, we examined the effect of AST on PI3K/AKT and NF-κB in T-HESCs. We found that the expression of p-AKT, p-NF-κB, and BCL2 was significantly decreased in the AST treatment group compared with the model group (Fig. 10A-C). Meanwhile, there was no significant difference in the expression of these proteins after AST treatment alone. These results indicate that AST suppresses TGF-β-induced fibrosis through AKT, NF-κB and BCL2.

## Discussion

IUA is a common clinical condition in gynaecology that can cause uterine infertility and miscarriage. With the high incidence and recurrence rates of IUA, the treatment of IUA is still challenging. In our previous study, we found that SCL was effective in alleviating by inhibiting endometrial fibrosis and inflammation [16]. There are many studies suggesting that inflammation and fibrosis of the endometrium play a key role in IUA. However, the mechanisms underlying the bioactive compounds and anti-IUA therapeutic effects of SCL remain unclear. Therefore, we used network pharmacology to predict the potential mechanisms and targets of action of SCL in IUA.

We selected 12 active compounds of SCL for IUA based on the criteria recommended by the databases based on OB ≥ 30% and DL ≥ 0.18. According to their degree values, the top 6 compounds in SCL were AST, kaempferid, isoengelitin, beta-sitosterol, diosgenin, and taxifolin. Among these, it has been realized that the ingredients of SCL may be have a therapeutic effect on IUA. For example, kaempferol affects the process of migration and invasion in endometriosis [22]. Beta-sitosterol exhibited an oestrogen-like effect, which was reported to reduce inflammation and fibrosis in the endometrium [23]. Taxifolin can alleviate ovarian damage from cisplatin [24]. Especially, in our results, AST was the more important key component with high degree and good binding energy. A series of studies have shown that AST inhibits fibrosis and inflammation in a variety of tissues, which similar etiology to IUA [25–28]. In addition, our previous research found that SCL could inhibit endometrial fibrosis and inflammation. Interestingly, we also found that AST was the most abundant component in SCL. Consequently, we focused on AST as we hypothesized that it is likely to play a key role in inhibiting the progression of IUA. In summary, the combination of results and literature suggests that the screened compounds may be beneficial in the treatment of IUA. Particularly, AST is the more potential compound in inhibiting inflammation and fibrosis. Therefore, the six compounds, especially AST, may act as a therapeutic agent for IUA by inhibiting endometrial inflammation and fibrosis.

Screening the data by bioinformatics assay, 15 core targets were identified. Among these, AKT, MTOR, VEGFA, CASP3, BCL2, MMP9, RELA (NF-κB p65) were associated with the pathogenesis of IUA [29–33]. Interestingly, a series of studies have shown that AST regulates the expression of CASP3, AKT, BCL2 and NF-κB in other disease models [33–35]. These proteins have high degree values in the PPI network, which means they could be potential targets for SCL treatment or the prevention of IUA. Furthermore, AST could be the potential active compound.

GO results analysis indicated that the effect of SCL in IUA is related to inflammation, autophagy, apoptosis and signal transduction. These biological processes have been verified in the study of the mechanism of IUA. It is widely believed that endometrial fibrosis is the pathological phenotype of IUA, with inflammation and autophagy being involved in this process [29, 36]. Furthermore, recent studies have found that apoptotic bodies appear to be a viable treatment option for IUA [37].

KEGG pathway analysis suggested that SCL may play an anti-IUA role by regulating PI3K/AKT and MAPK signalling pathways. The main compounds of SCL, beta-sitosterol, kaempferol, taxifolin and AST, are associated with both pathways. For instance, beta-sitosterol significantly inhibits proliferation and migration by blocking the EGFR/MAPK signalling pathway [38]. Kaempferol exhibits significant anti-inflammatory effects and regulates VEGF/AKT pathways [39]. Taxifolin inhibited inflammatory response and hepatocellular regeneration via PI3K/AKT and MAPK signalling pathways [40]. AST suppresses fat accumulation via the activation of AMPK and partially ameliorates necroptosis mediated by PI3K/AKT activation [35, 41]. NF-κB is a downstream protein of AKT. AST attenuated LPS-induced myocardial injury and inflammation by inhibiting TLR4/ NF-κB pathway [42]. Besides, many studies have demonstrated the development of IUA is also related the PI3K/AKT and MAPK pathways. Activation of the PI3K/AKT pathway promoted cellular fibrosis and IUA development [43]. AKT/NF-κB pathway and MAPK/ERK-MTOR pathway also involved in endometrial epithelial-mesenchymal transition and IUA [29, 44]. Briefly, the main pathways enriched in this study were the MAPK and PI3K/AKT pathways, which are generally involved in inflammation and fibrosis. The results are consistent with the literature. It suggests that SCL may have a critical role in treating IUA via the signalling pathways. Furthermore, AST, a more important compounds of SCL, acts in other diseases by regulating the PI3K/AKT pathway, AMPK and NF-κB [33, 35, 41]. Therefore, it was further hypothesized that AST might a potential treatment for IUA by acting through the PI3K/AKT pathway and NF-κB.

In this study, we analysed the chemical components and key targets in SCL with the help of PPI and KEGG analyses. According to the results, we identified six components (kaempferol, isoengelitin, AST, beta-sitosterol, diosgenin, and taxifolin) and 10 targets (RELA, AKT, MTOR, CASP3, BCL2, EGFR, VEGFA, HIF, MMP9, and TP53) that were used for molecular docking. The docking results showed binding energies between − 15.72 and − 6.22 kcal/mol, which indicates a strong binding activity between these active ingredients and targets. The binding energies of MMP9, RELA, VEGFA, EGFR, AKT and BCL2 were the lowest. AST, beta-sitosterol and diosgenin have good binding activity to these targets, suggesting that they may be useful in the treatment of IUA.

Based on KEGG, GO and molecular docking analyses, AKT, BCL2, and RELA (NF-κB p65) may be the main targets of SLC for IUA treatment. Furthermore, AST is the most likely molecule in SCL to be a treatment for IUA. Therefore, to validate our predicted results, western blot was used to further determine whether AST affected the protein expression of AKT, NF-κB and BCL2 in vitro. We found that AST significantly reduced the overexpression of these proteins induced by TGF-β in T-HESCs. Overall, the results suggested that the anti-IUA effect of SCL is associated with AST and directly related to the PI3K/AKT/NF-κB signalling pathway.

There are limitations of this study. The sources of the data were dependent on known databases and the literature. Therefore, a large part of the reliability of data depends on the quality of the literature and the updating of the databases. Although AST is the most abundant component of SCL, the role of other components in IUA should not be overlooked. Accordingly, further experimental validation is also worthwhile for other components. In addition, accounting for the differences between in vitro and in vivo conditions, it is necessary to include animal and clinical trials for further studies to confirm the mechanisms.

## Conclusions

In conclusion, this is the first time that network pharmacology, molecular prediction, molecular docking, and in vivo experiments have been used to explore the mechanisms and potential targets of SCL in IUA. These bioinformatic results impliy that the molecular mechanisms of SCL in IUA are mainly related to the PI3K/AKT signalling pathway, NF-κB and BCL2. In addition, we found that AST is most likely the active ingredient for the treatment of IUA, and the results of in vivo experiments verified the above points.

Overall, we discovered the mechanism of action and targets of SCL for the treatment of IUA. These findings will provide guidance for the application and advancement of SCL in the treatment of IUA.

### Electronic supplementary material

Below is the link to the electronic supplementary material.

Supplementary Material 1

Supplementary Material 2

Supplementary Material 3

Supplementary Material 4

Supplementary Material 5

Supplementary Material 6

Supplementary Material 7

Supplementary Material 8

Supplementary Material 9

Supplementary Material 10

## Data Availability

All data from this study are available from the corresponding author on reasonable request.

## Abbreviations

**IUA**: Intrauterine adhesion
**D&C**: Dilation and curettage
**TACR**: Transcervical resection of adhesion
**SCL**: Smilax china L.
**TCMSP**: Traditional Chinese Medicine Systems Pharmacology Database
**DL**: Drug-likeness
**OB**: Oral bioavailability
**PPI**: Protein-Protein Interaction
**GO**: Gene ontology
**KEGG**: Kyoto Encyclopedia of Genes and Genomes
**AST**: Astilbin
