# Supplementary material for: Mechanism of Smilax china L. in the treatment of intrauterine adhesions based on network pharmacology, molecular docking and experimental validation
Source: BMC Complement Med Ther. 2024 Apr 5;24:150. doi: 10.1186/s12906-024-04414-4 (PMC10996135; doi:10.1186/s12906-024-04414-4)
Supplement: Supplementary file 10 — Supplementary Material 10 [file 12906_2024_4414_MOESM10_ESM.docx]

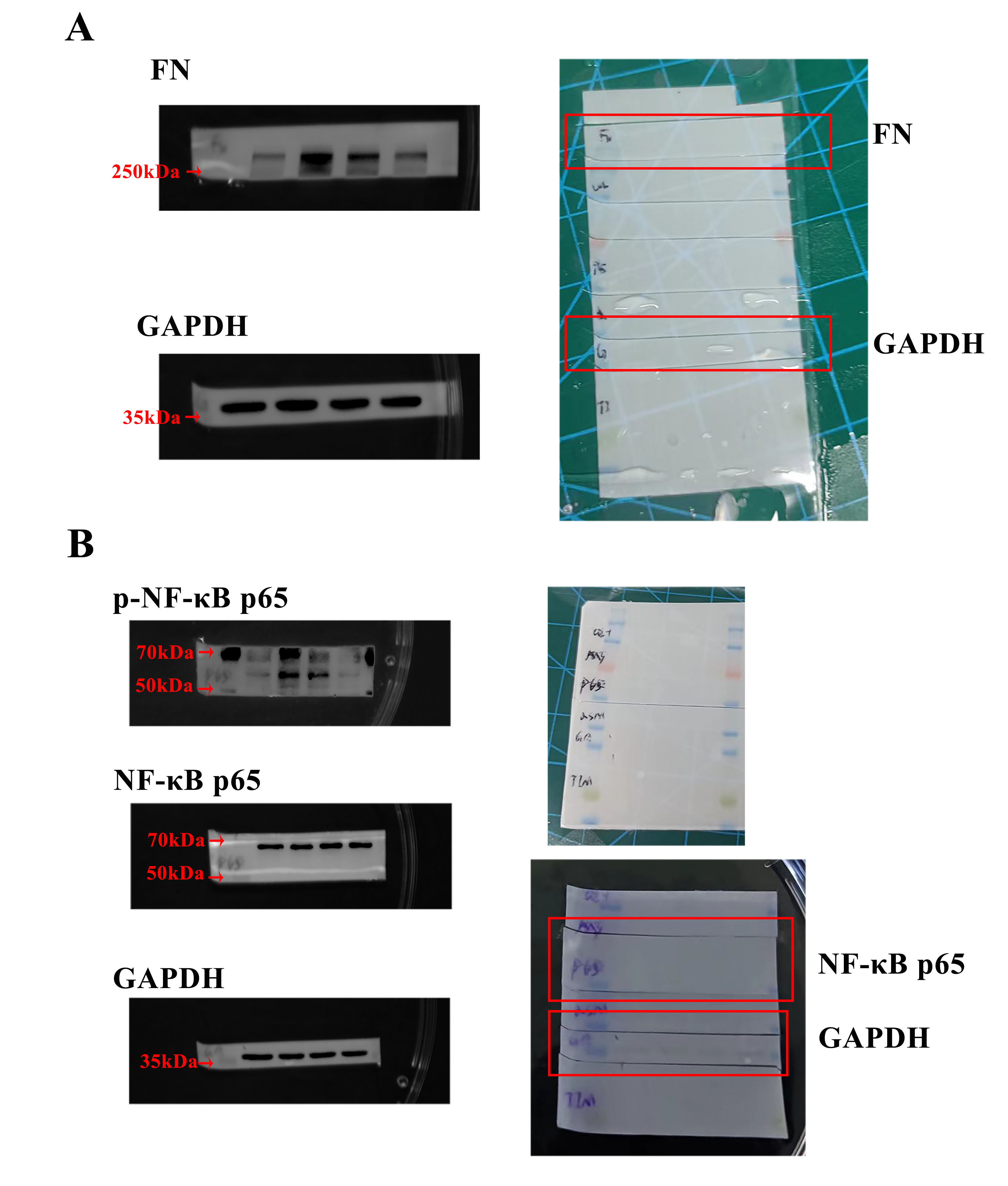


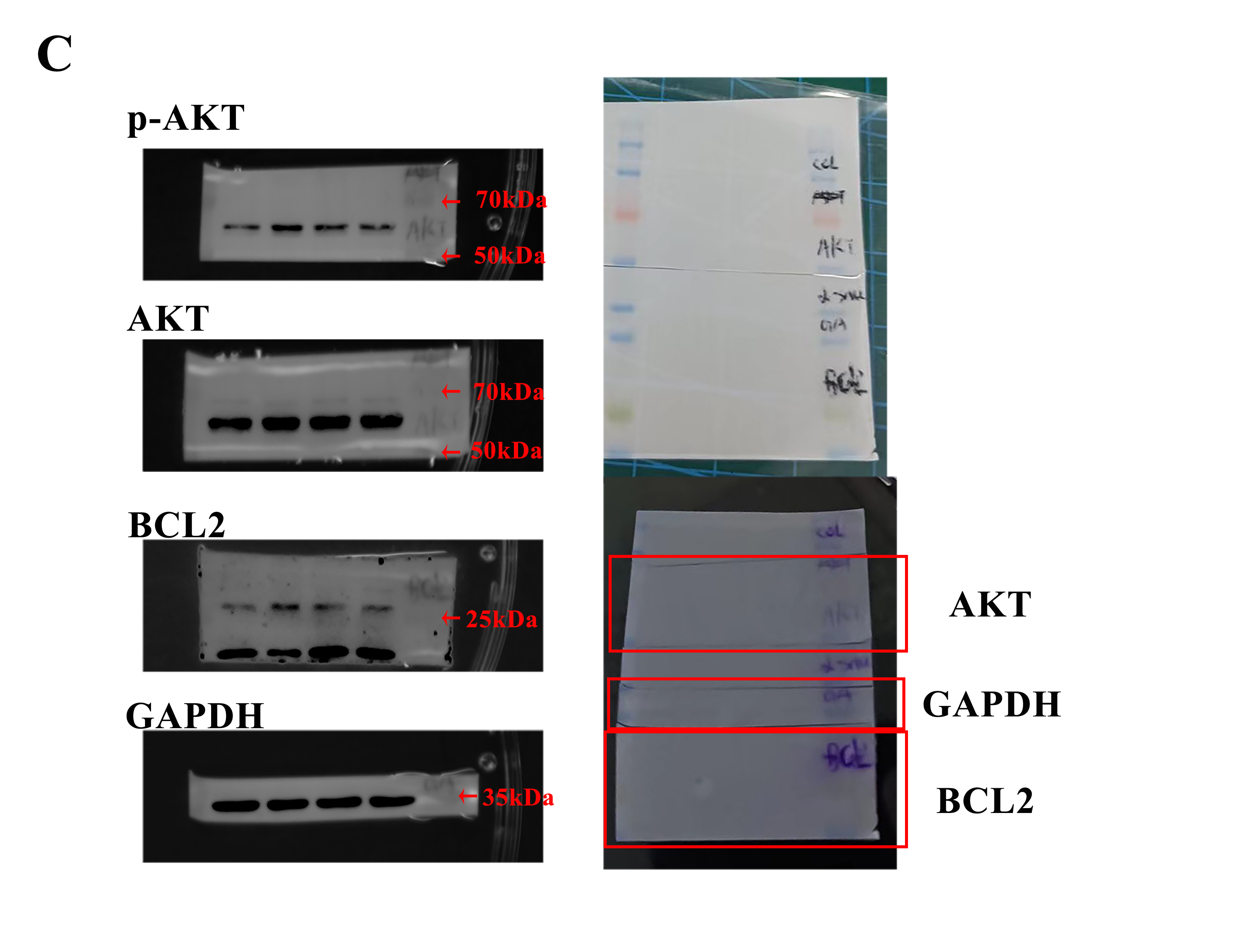


Figure S1.Western Blot original images. The figure represents a Western Blot (WB) where we used the technique of cutting blots and then hybridizing with antibodies. It is worth noting that the whole membrane on the right side of the figure is able to demonstrate that the destination bands all originate from the same membrane. In addition, the cropping method employed does not disrupt the integrity and authenticity of the visual presentation. The lanes for this WB are, from left to right, NC, TGF-β, TGF-β+AST, AST. AST indicates astilbin.
